# Agrobiodiversity-Based Landscape Design in Urban Areas

**DOI:** 10.3390/plants12244121

**Published:** 2023-12-10

**Authors:** Rita Biasi, Elena Brunori

**Affiliations:** Department for Innovation in Biological, Agri-Food and Forest Systems (DIBAF), University of Tuscia, via S. Camillo de Lellis snc, 01100 Viterbo, Italy; brunori@unitus.it

**Keywords:** biodiversity conservation strategies, ecosystem services, food-forests, food policies, planting design, Mediterranean cities, one health approach, urban agriculture, *Vitis vinifera* L., well-being

## Abstract

Agrobiodiversity represents a system of biological organisms that contribute to agri-food production. In a context marked by a significant loss of food-relevant species and a reduction in their genetic diversity, the adoption of strategies to preserve and enhance the diversity of genetic resources that support and complement agricultural production has become a global challenge. Many sustainable development strategies outlined in recent years directly and indirectly attribute a crucial role to agrobiodiversity in meeting food needs, ensuring food system security, promoting food justice, and enhancing well-being in modern living environments. This contribution aims to analyze the process of knowledge and awareness that has led many cities to plan their urban development by investing in the agricultural matrix and to address the design of open spaces with agricultural biodiversity.

## 1. Introduction

Agrobiodiversity represents a highly complex biological system with a recognized role not only as a direct source of food but also as a frequently overlooked contributor to environmental quality and well-being. According to the FAO’s definition [1], agrobiodiversity encompasses “The variety and variability of animals, plants, and micro-organisms that are used directly or indirectly for food and agriculture, including crops, livestock, forestry, and fisheries. It comprises the diversity of genetic resources (varieties, breeds) and species used for food, fodder, fiber, fuel, and pharmaceuticals. It also includes the diversity of non-harvested species that support production (soil micro-organisms, predators, pollinators), and those in the wider environment that support agro-ecosystems (agricultural, pastoral, forest, and aquatic), as well as the diversity of the agro-ecosystems”. Therefore, agrobiodiversity represents an integrated living system with a multi-scalar dimension, ranging from ecosystems to genes.

In recent years, there has been a growing interest in agrobiodiversity and its utilization due to increased awareness of the environmental benefits, in addition to its role in food production. International, national, and local strategies have contributed to orienting food policies, agricultural production, rural and urban area planning, design, and management. The UN 2030 Agenda, with Sustainable Development Goals (SDGs) 2 (Zero Hunger), 13 (Climate Action), 15 (Life on Land), and 11 (Sustainable Cities), emphasizes the pivotal role of agriculture in achieving these objectives. Furthermore, the European Green Deal, with its specific strategies, such as Biodiversity 2030, Farm to Fork, and Soil Strategy [2,3,4], has highlighted the use of agrobiodiversity for human health and environmental quality. The Farm to Fork strategy, in particular, focuses on promoting actions for sustainable food production, processing, distribution, consumption, and reducing food loss and waste. Recently, the New European Bauhaus (NEB) strategy [5], which aims to guide sustainable, inclusive, and aesthetically pleasing architectural practices, has given new insight into the use of agrobiodiversity in architectural design. This implies not only the integration of urban agriculture into buildings, like building-integrated agriculture (BIA), which encompasses practices like rooftop and vertical farming [6], but also broadens the food environments through the implementation of architectural strategies and innovative solutions for a stronger citizen connection to food and self-provisioning through multifunctional, nature-based solutions, such as urban gardens integrated with parks, edible trees and bushes, and edible green infrastructure.

The One Health approach, promoted by the World Health Organization, emphasizes a systemic approach to human, animal, and environmental health and also recognizes the key role of agrobiodiversity [7,8], as also highlighted by the FAO. Food safety is considered “a core tenet” of One Health, and it underscores the transformation of today’s food systems to increase biodiversity, ecosystem services, environmental and soil health, and food security. The sustainability of food production, therefore, requires the preservation of existing agricultural landscapes or the sustainable design of new ones, given the constant decrease in agricultural land [9] together with the increased demand of food for humans, animals, and biofuel.

## 2. Landscape-Related Agrobiodiversity Conservation Strategies

The current status of agrobiodiversity is one of extreme impoverishment. The richness of species of food interest was documented by the geneticist Vavilov (1996) [10], who reported the existence of 11 centers of origin for species of food interest, where wild species were domesticated and spread worldwide, mainly due to migration and expeditions. Out of over 6000 edible species, only 200 significantly contribute to global food production, and 9 of them make up 66% of the cultivated species [1,11]. The Mediterranean basin has not escaped this negative trend, even though rich agrobiodiversity has accumulated over time (Table 1). This area, due to its geography, is both a center for biodiversity flows and a region where the diversity of physiographic traits, along with the diversity of local history and culture, has given rise to genetic resource variability through natural genetic mutations and/or breeding and clonal selection by farmers or researchers, resulting in a hotspot of biocultural diversity. Dozens of food species and hundreds of varieties were cultivated until the mid-20th century, when the advent of industrialized and specialized agriculture drastically reduced the genetic resources that underlie agri-food production. In contrast, the genetic resources at the bases of the Mediterranean diet, recognized as intangible heritage by UNESCO since 2010 [12], form the basis of a healthy diet and of the preservation of local rural culture and landscapes. The richness of this indigenous agrobiodiversity is threatened by numerous factors [11,13,14], and genetic erosion remains high despite efforts to conserve it.

The preservation of indigenous agrobiodiversity is a contemporary challenge, particularly due to its strategic contribution to mitigating and adapting to climate change. Local landraces, co-evolved with their native environments, represent varieties with a high capacity to tolerate biotic and abiotic stresses, resulting in crop stability and reduced input requirements [16]. They provide a reservoir of valuable genes for selecting new cultivars that enable more resilient agriculture. In addition to well-established strategies for *ex situ* biodiversity conservation, such as core collections in open fields by research institutions or in vitro germplasm conservation, two other strategies allow for the preservation of precious local genetic resources along with the safeguarding of unique landscapes. The *in situ* on-farm conservation strategy promotes the maintenance of local genetic resources within agroecosystems, as individual plants or small orchards and fields, and the related complex landscapes [14]. The European Common Agricultural Policy (CAP) and the local agricultural policies can support farmers in this role as guardians of species, cultivars, and traditional landscapes, as the link between autochthonous agrobiodiversity and conservation of traditional agricultural landscapes of high cultural and environmental value is well established [17]. Furthermore, a key strategy involves also preserving genetic diversity in the centers of origin of specific food species, which can serve high aesthetic and environmental functions. The forests of wild apple trees in the Tien Shan region of Kazakhstan were awarded the International Carlo Scarpa Prize for Gardens (XXVII Edition, 2016), along with other landscapes, i.e., the Rose Valley and the Red Valley in Cappadocia (XXXI Edition, 2020–2021) [18], recognizing these natural ecosystems, coexisting with residual agro-ecosystems based on local genetic resources, with unique value and cultural functions.

Finally, cities themselves are, nowadays, playing a key role in preserving precious agrobiodiversity through the different typology of urban agriculture, mainly home-based gardening, or community-based and other shared gardening, that are frequently hotspots of local genetic resources at risk of erosion. Nonetheless, in this paper, the city’s contribution to this biological function is argued, referring to the planting design of open spaces. 

## 3. Agrobiodiversity and Environmental Quality

The quality of living environments is a pivotal challenge for sustainable development. This requires the use of nature-based solutions (NBSs), and agrobiodiversity can play a crucial role in addressing these challenges. Sustainable agriculture, whether conservative, organic, or based on agroecological principles, not only ensures food security and nutrition but also contributes to environmental quality [19,20,21], thus assuming a public and social role. However, there is a concerning global trend of agricultural land erosion, with cropland decreasing, especially in industrialized countries. Projections for 2030 [9] suggest a substantial reduction in the near future. Drivers and consequences of agricultural land erosion have been linked to soil consumption, climate change, socio-economic factors, geographical and environmental characteristics, and the consequences include the loss of multiple ecosystem services, including soil biological fertility, hydro-geomorphological stability, and landscape quality [22]. In both rural and peri-urban areas, agricultural abandonment often leads to the loss of precious traditional ecological knowledge associated with traditional agriculture, which has negative externalities on culture and the environment. The lessons from traditional farming, particularly in sustainable soil fertility management and water resource use, mainly by developing systems for managing scarce water resources in arid environments, are significant, especially in the context of carbon farming and conservative agriculture as responses to climate change mitigation and adaptation. Notably, the agroecological approach adopted for agro-ecosystem management in traditional agriculture is seen as a pathway to creating a sustainable landscape [23].

The ecosystemic functions of agriculture are valuable not only for rural areas [24,25,26] but also for urban environments [27,28,29], which are becoming elective places to live. The challenge for cities to transition to biocities is based on NBSs, including investments in natural capital, like urban forests, as well as agroforestry and agricultural areas [11,30]. However, species and genotypes, to effectively fulfill their environmental functions, need to adapt to urban environments, ensuring essential physiological functions, like photosynthesis and transpiration, which are fundamental for carbon storage, urban microclimate mitigation, and soil functionality maintenance. As a result, it is essential to continue testing suitable genetic resources that demonstrate resistance and resilience in urban settings. Many studies are evaluating the suitability of herbaceous and woody ornamental species, as well as food species, primarily concerning their adaptation to abiotic stresses, including multiple summer stressors [31,32].

In this context, agrobiodiversity assumes a special significance, not only in urban planning [33] but also in urban landscape design [34]. This paper will develop this topic, particularly in relation to Mediterranean cities. 

## 4. Designing New Food Landscapes

### 4.1. Conceptual Framework

The utilization of food species in landscape design is gaining significance due to the widely acknowledged multifunctionality of productive landscapes, both in rural and urban settings. Furthermore, agrobiodiversity plays a crucial role in landscape design at various scales, ranging from the broad landscape scale to site-specific applications. 

An emerging trend in the agronomical sciences and related disciplines is to incorporate the landscape dimension into the design and management of agro-ecosystems [35,36,37]. Previously, the agricultural landscape was often viewed primarily for its aesthetic qualities and, therefore, considered non-essential to the agronomic technique. However, in recent years, the agricultural landscape, as a result of transforming the natural landscape for agricultural production, has gained structural and functional significance. Incorporating the landscape dimension into various agri-food production chains has the potential to enhance the safeguarding of local foods and food-related local resources [38]. Aligned with the objectives of sustainable development strategies (see Section 1), particularly the preservation of biodiversity in terms of species and ecosystems, the conservation of soil health and functionality, and the optimization of carbon storage processes, there is a growing recognition for the need to redesign or restore the agricultural landscape to enhance its complexity in terms of shapes and land use [39,40,41,42,43]. These are landscape functional traits that have been lost in intensive agricultural systems [35]. Nonetheless, even in areas of intensive and specialized crop cultivation, the agricultural landscape may incorporate natural remnants, small woodlands, tree-lined paths, out-of-forest trees, floral strips, and riparian vegetation, all of which serve as ecological functional components and, therefore, highly encouraged by the European CAP. Additionally, beyond these functional aspects, the aesthetic component resulting from the design of the agricultural land [36] is increasingly viewed as an opportunity to enhance the competitiveness of farms and territorial products. Consequently, landscape quality may now be considered an integral part of the overall quality of agri-food products.

**Figure 1:**
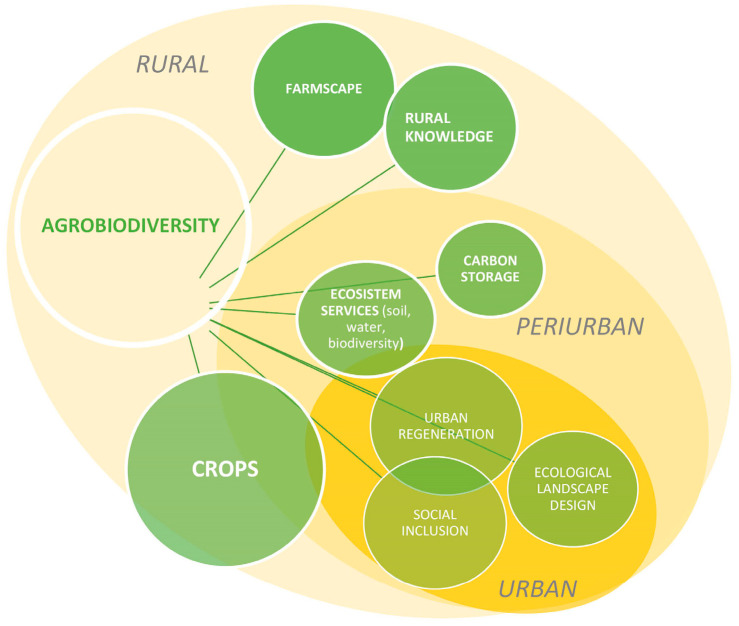
Schemes of the main services derived from the use of agrobiodiversity in rural, peri-urban and urban areas. Circles represent the main functions that agrobiodiversity exerts in each ambit. Overlapping between circles represents interconnected functions; overlapping between landscape types represents shared functions among rural, peri-urban or urban areas. (Credit: R. Biasi).

Cities play a crucial role as essential collaborators in realizing sustainable global agriculture, fostering health, and advancing climate and biodiversity objectives. Cities can strengthen their contribution through climate adaptation strategies that actively involve health considerations, prioritize nature-based solutions, and place increased emphasis on food and nutrition [44]. Ecological landscape design and planning involving agrobiodiversity may serve as a tool for a low-carbon strategy. In the urban context, agricultural land plays a crucial role in providing various ecosystem services, particularly when it follows the agroecological approach [19,45,46]. The expansion of cities has predominantly occurred at the expense of the surrounding countryside, and remnants of cropland are common in the transitional urban–rural areas. Bridging the gap between these two ecosystems, the city and the countryside, presents a significant challenge for the sustainable development of metropolitan areas [11]. In this context, productive landscapes within and near the city contribute to the transformation of food systems towards a strategy of sustainable food distribution and consumption, often referred to as proximity agri-food production, guided by local food policies [7]. The implementation of cities’ food policies has increased the demand for cropland in many metropolitan areas, with the aim of not only establishing short food supply chains but also creating spaces for food education, food justice, and social cohesion [47]. However, the role of productive land as a component of the city’s green infrastructure often goes beyond these functions. The strategy of designing patches of cultivated land in the open spaces of the city, including underutilized and interstitial empty areas, verges, as well as in urban parks and gardens, aims to create a “continuous productive urban landscape” (CPUL) [48] that serves as sustainable green infrastructure. This approach is not new; however, the affirmation of principles related to ecological and low-carbon design has reinforced the trend of adopting nature-based solutions (NBSs) through urban agrobiodiversity-related projects [49,50]. Thanks to these multiple benefits, the design of productive land can also be used to regenerate and restore degraded areas. Restoring a degraded place with a vegetational intervention that requires systematic land care and community engagement, as the agricultural practice implies, represents a challenging yet sustainable strategy for urban development. The multifunctionality of agrobiodiversity in the different landscape ambits, i.e., the rural, peri-urban, and urban ecosystems, is reported in Figure 1. 

Achieving the objective of agrobiodiversity multifunctionality depends on the balance of agroecosystems and the vegetative and productive behavior of food species, closely related to plant–soil–atmosphere interactions. The urban environment, in general, is highly stressful for plants, and this is especially true for food plants. The climate in many metropolitan cities is well known to induce multiple stresses, such as light pollution, heat waves, aridity, and storms. In contrast, urban soil characteristics are much less known, particularly in terms of agro-pedological suitability, which is based on physical–chemical and biological fertility [51]. Furthermore, habitat patches in the city are small, heterogenous, fragmented, suitable, or unsuitable to biological systems. Therefore, identifying suitable urban spaces for healthy urban peri-urban agriculture (UPUA) is becoming an important challenge [52,53]. The availability of detailed land capability maps can aid in identifying not only the vulnerable land for implementing the city’s agricultural matrix but also areas suitable for preservation, not only from soil consumption but also from urban afforestation. 

**Figure 2:**
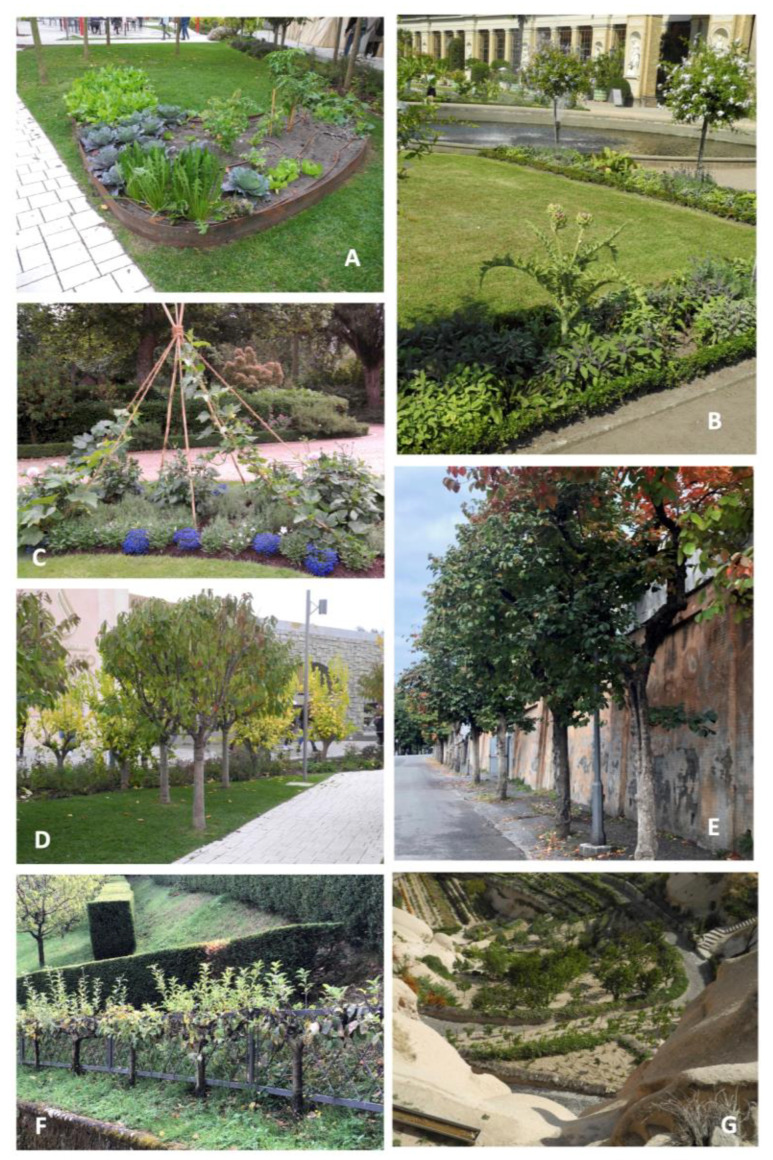
Species of food interest are used for flowerbeds design (**A**–**C**), small-scaled urban orchards (**D**), tree rows (**E**), espaliers in historical gardens (**F**), urban open space design (**G**). (**A**,**D**) flowerbed of vegetables (**A**) and promiscuous fruit orchard (**D**), Expo 2015 area, Milan Italy; (**B**), flowerbed of vegetables and aromatic plants, Sans Souci Park, Postdam Germany; (**C**), Cucurbitaceae Collection, Jardin des Plantes, Lyon France; (**E**) tree row of *Diospyros kaki*, Villa Borghese gardens, Rome Italy; (**F**) *Malus domestica* tree espalier, Villa Bardini, Florence Italy; (**G**) horticultural crops, Argos Hotel garden, Üçhisar Turkey (Photos credit: R. Biasi).

As we delve further into the urban application of agrobiodiversity, the aesthetic aspect becomes increasingly important. Many food species are now being considered for multiple purposes in urban tree lines, parks, green areas, and flowerbed designs (Figure 2). This trend often involves replacing traditional ornamental species, mainly those that are allochthonous and more recently introduced in Mediterranean environments, which has allowed them to become invasive, like *Carpobrotus edulis*, *Ailanthus altissima*, and *Ligustrum lucidum*. Several reasons may drive this shift, including: (i) adaptation to the environment—autochthonous (native) food species are better adapted to the local environment, which can enhance their performance and resilience; (ii) reduced allergenic potential—native food species may have a lower allergenic potential compared to some non-native ornamental species; (iii) preservation of local food and tradition—the use of native food species in urban landscapes can serve as a reminder of the importance of local food and rural traditions; (iv) aesthetic appeal—food species, including fruit trees, are appreciated not only for their utility but also for their aesthetic qualities. The presence of fruit trees in gardens is historically well documented [54]. Furthermore, contemporary breeding programs for many food species have selected genotypes with enhanced aesthetic traits. These traits may include abundant and recurrent flowering, diversified fruit shapes and colors, and specific tree architectures and sizes, so that, nowadays, fruit trees, fruiting vines, and fruiting bushes have become integral components of ornamental nursery products. In parallel, ongoing research seeks to identify solutions and architectural devices that enhance the aesthetic function of vegetable gardens, orchards, and flowerbeds in urban environments [55,56,57]. Edible and ornamental plants, blending visual appeal with food utility, can serve as a valuable resource in the design of urban landscapes. Given the ongoing challenges of climate change and the need for adaptation and mitigation, the identification of resistant genotypes is a prerequisite for developing resilient UPUA and urban landscape projects. This approach not only supports food production but also contributes to the visual and cultural aspects of urban landscapes that can preserve, promote, and exhibit autochthonous agrobiodiversity toward a citizens’ food and environmental education. 

### 4.2. An Emblematic Food Species for the Urban Landscape Design

Many Mediterranean food species t historically represent constitutive elements of the urban landscape and are becoming more and more widely employed in urban planting design. *Olea europaea* and *Citrus* spp. (mainly *C. aurantium* and *C. sinensis*) are so widespread, both in the Mediterranean rural and urban areas, that their presence constitutes a unifying landscape matrix. Olive and orange trees are elements of urban open spaces, city’s tree rows, and a myriad of balconies and terraces hosting single trees. Owing to climate change, these new urban olive and citrus landscapes are moving northward and to higher altitudes. Nonetheless, among the food species largely used in urban landscape design, one appears to prevail owing to its high multifunctionality, the *Vitis vinifera* L. This species, being one of the first cultivated in the Mediterranean [15], has been present in the city since ancient times. From its introduction in the Mediterranean, in the Roman and Etruscan territories, vineyards were part of the urban and peri-urban landscape, mainly for their productive functions, i.e., grape provision and vinification. Still, vineyards represent one resilient land use in the urban–rural space of many Mediterranean cities, regardless of soil consumption, and the city’s enlargement, owing to high-quality products of high added value and the recognized provision of important environmental services, from biodiversity and ecosystems’ conservation to carbon storage [58,59,60]. Urban vineyards, at the same time, function as crucial patches for the persistence of native animal biodiversity in the urban landscape [61], becoming representative of key land uses, fostering structural and functional ecological connectivity within the city. Furthermore, identity elements of the sites, in many historical and archeological areas, are preserved or have redesigned vineyards, for retaining or recovering the *genius loci* of the historical place, in accordance with the local agricultural traditions [62]. The phenomenon of urban vineyards is becoming a challenge in land use for the cities’ multifunctionality [63].

**Figure 3:**
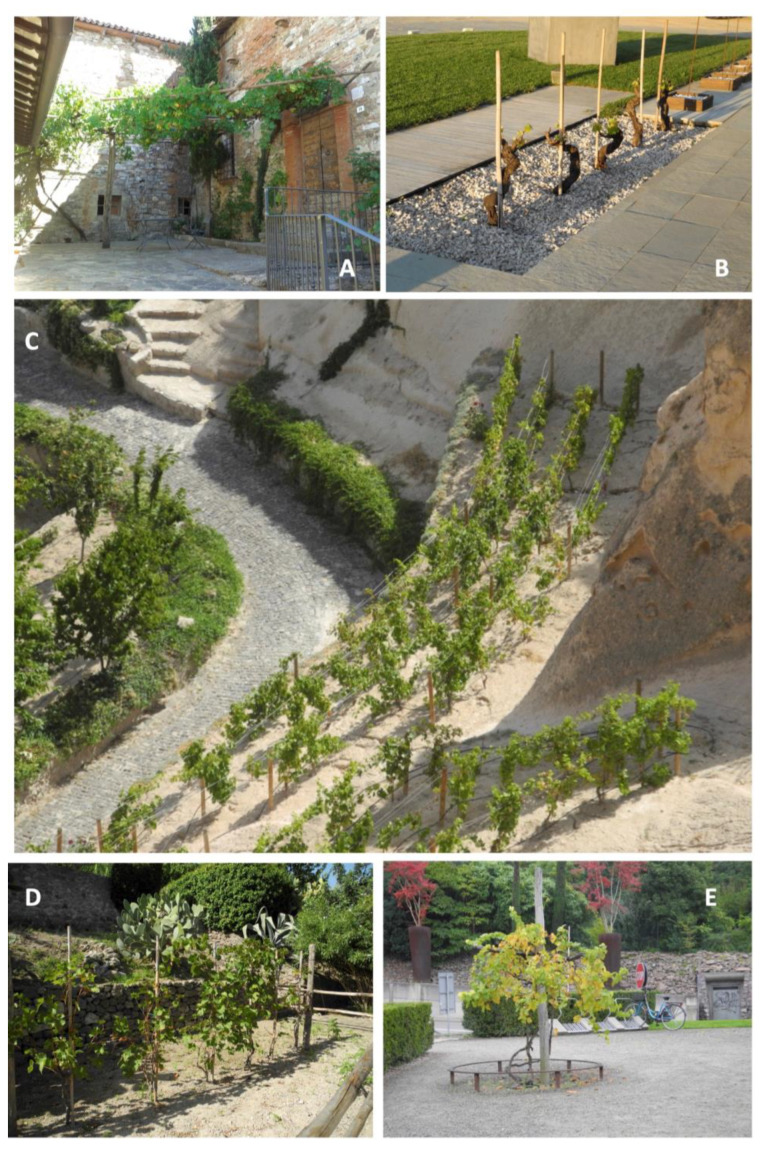
The multifunctional use of *Vitis vinifera* L. in urban contest and multi-scaled landscape design example; from single vine architectures (**A**,**E**), to vine rows (**B**) and small-scaled vineyards (**C**,**D**). (**A**), San Venanzo, Umbra Region Italy; (**B**) Surrau Winery, Sardinian island Italy; (**C**) Argos Hotel garden, Üçhisar Turkey; (**D**) gardens of Aragonese Castel, Ischia island Italy; (**E**), parking area, Meran Italy (Photos credit: R. Biasi).

The versatility of *Vitis vinifera* is evident in its use across various scales and forms in urban landscape projects (Figure 3). This species, with all of its genetic diversity, is employed in multifunctional urban designs, ranging from individual vines to extensive orchards. The plasticity in the growth habit of *Vitis vinifera* and its high adaptability to many environments make it suitable for various urban design applications. It can be integrated into living architectural elements, used to create tree-lined streets, incorporated into flowerbeds, and featured in home gardens. Additionally, new small-scale urban vineyards serve multiple purposes, such as ex situ agrobiodiversity conservation, providing opportunities for citizen food education, and promoting wine tourism by preserving local grape-wine culture and traditions [31,64]. In addition to this function, grapevine rows are frequently used in planting design just for using better-adapted genotypes to the urban environment and habitats. This use of *Vitis vinifera* also helps counteract the botanical pollution caused by invasive and alien species. 

By employing *Vitis vinifera* in open space design, urban planners and landscape architects can create multifaceted, punctual, linear, or extensive landscapes. These designs are well suited for the fragmented, residual, and underutilized spaces typical of urban environments, making them a valuable addition to the urban fabric.

### 4.3. Toward Urban Landscape Complexification with Agroforestry Design

Species association plays a crucial role in designing functional farming systems based on biodiversity. This principle is not limited to traditional agricultural landscapes, with agroforestry being an age-old practice [65], but is also being integrated into intensive and specialized agricultural systems with the aim of achieving sustainable intensification. Nature-Based Farming Solutions (NBFSs) are instruments for productive landscape quality and resilience [23,66]. The complexity of biodiversity within landscapes is seen as a strategy to enhance the quality and resilience of cities.

Cities’ quality is mainly attributed to urban forests and urban green infrastructures. As cities transition towards sustainability, inclusivity, and resilience, there is a growing emphasis on the expansion of NBSs based on urban forestry. Urban forests are seen as fundamental components of modern and future biocities, contributing to the well-being of urban areas and their inhabitants [67,68]. They offer numerous benefits, including improved air quality, enhanced biodiversity, reduced urban heat island effects, and recreational spaces, making cities more livable and sustainable. 

Nonetheless, agroforestry systems are becoming increasingly important elements within the urban environment [69,70]. They serve as multifunctional spaces that contribute to various aspects of urban life. These systems are designed to support local food policies and are characterized by their complexity, as they involve the interplanting of forest tree species with those of food interest. This approach results in specialized crop associations, riparian corridors, edible windbreaks, and even full-fledged food forests [65]. Urban food forest’s role, when incorporated into public spaces, is as a supplier of both food and public engagement, emphasizing the design of spaces, facilities, and events focused on the suitability of food systems [70,71,72]. The Picasso Food Forest in Parma, Italy, is a representative edible landscape project [70], both from a technical and goals perspective. Almost 6000 m^2^ of an urban neglected space recovered through the serial planting of multilayered vegetation, after soil regeneration through the employment of nitrogen fixing cover crops. Planting design was based on the employment of large forest trees (*Acer* spp.), small fruit trees (like *Prunus armeniaca*, *Prunus salicina*, *Prunus persica*, *Malus domestica*, *Punica granatum*), shrubs (like gooseberries, raspberries, currants), an herbaceous layer (of aromatic plants, herbs and vegetables), and, finally, living mulch by strawberry plants, oregano, and clover, together with tubers growing in the rhizosphere following the principle of arrangement of layers, aiming at space use optimization and biological balance based on complex relations among biological systems. 

Urban agroforestry systems take various forms and can be found in different locations, ranging from private home gardens to public spaces. The multifunctionality of these edible urban green infrastructures has been recognized in initiatives like the NEB strategy [5]. They serve as nature-based solutions that contribute to biodiversity conservation, enhance ecosystem services such as pollination and soil health, promote social inclusion through community agroforestry systems, and support food justice initiatives, becoming, therefore, key new landscapes for urban design [73,74,75]. These systems aim to provide ecological and social benefits that improve the well-being and global health of urban residents. 

The integration of productive landscapes into future biocities represents a significant challenge but also an opportunity to enhance the quality of contemporary habitats. It allows citizens to reconnect with nature and access healthy and sustainable food sources, contributing to their overall well-being and global health. 

## 5. Concluding Remarks 

The role of cities, elective places for contemporary living, is of strategic importance in maintaining agrobiodiversity and biodiversity-related ecosystem services. Urban food systems are central to achieving sustainable goals, aligning with initiatives like the UN Food 2030 and the European Farm to Fork strategies. These initiatives stress the importance of providing a sustainable food environment, assuring healthy, safe, and high-quality food for all while promoting resource efficiency and sustainability.

Strengthening agrobiodiversity in cities and, therefore, expanding the food environment are vital for developing more sustainable food systems based on local production chains. Urban and peri-urban areas already play a role in supporting food production systems by integrating agrobiodiversity at various levels in landscape planning, design, and management, resulting in better connections between farmscape and open urban spaces.

Integrating agrobiodiversity within the urban tissue contributes to the preservation of natural capital in the variety of its nature. It may also serve as a strategy for urban regeneration and the revitalization of degraded or low-quality habitats. In fact, landscape architecture is increasingly considering the integration of the productive landscape into the city’s green infrastructure network, creating multifunctional nature-based solutions integrated with urban edible trees and bushes, urban food forests, parks, and home gardens.

The use of agrobiodiversity-based ecological design criteria, through the best use of soil to be productive, becomes a valuable tool for safeguarding soil health and functionality, counteracting land degradation. Future research efforts should be oriented toward the identification of the most suitable spaces for investing in agrobiodiversity, based on agro-pedological suitability (land capability), environmental characteristics, organizational, and social aspects. This approach could also facilitate the design of contemporary food landscapes based on species/genotypes capable of adapting to sensible, human-dominated areas and guide landscape design toward the definition of a holistic complex agroforestry system where urban forests become productive and nature-based farming solutions allow cropland to be more treed, for resilient and inclusive cities.

## Figures and Tables

**Table 1:** The times of recorded presence of fruit tree species in the Mediterranean (source: J. Janick, 2005 [15]). BC, before Christ; AD, Anno Domini; c., century.

Crop	Species	Center of Origin	Moment of Introduction
Fig	*Ficus carica*	Mediterranean	3000 BC
Grapevine	*Vitis vinifera*	Western Asiatic	5000 BC
Olive	*Olea europaea*	Mediterranean	700/600 BC
Pomegranate	*Punica granatum*	Western Asiatic	5000 BC
Apple	*Malus domestica*	Central Asiatic	1000 BC
Pear	*Pyrus communis*	Central Asiatic	1000 BC
Asian pear	*Pyrus pyrifolia*	Eastern Asiatic	20th c. AD
Almond	*Prunus dulcis*	South-western Asiatic	200/300 b.C.
Apricot	*Prunus armeniaca*	Central-western Asiatic	100 b.C.
Sweet cherry	*Prunus avium*	Central European	300 b.C.
Peach	*Prunus persica*	Chinese	300 b.C.
Kiwifruit	*Actinidia deliciosa*	Chinese	20th c. AD
Citrus fruits	*Citrus* spp.	Southern Asiatic/Chinese	11th c. AD

## Data Availability

Not applicable.
